# Zn-Ni metal-organic framework nanosheets@graphene oxide nanocomposite: A promising voltammetric platform for sensitive determination of doxorubicin

**DOI:** 10.5599/admet.3384

**Published:** 2026-07-08

**Authors:** Tahereh Kondori, Niloufar Akbarzadeh-T, Somayeh Tajik, Hadi Beitollahi

**Affiliations:** 1 Health Promotion Research Center, Zahedan University of Medical Sciences, Zahedan, Iran; 2 Department of Chemistry, University of Sistan and Baluchestan, P.O. Box 98135-674, Zahedan, Iran; 3 Research Center of Tropical and Infectious Diseases, Kerman University of Medical Sciences, Kerman, Iran; 4 Environment Department, Institute of Science and High Technology and Environmental Sciences, Graduate University of Advanced Technology, Kerman, Iran

**Keywords:** Cancer, differential pulse voltammetry, voltammetric sensor, real sample, synergistic interaction

## Abstract

**Background and purpose:**

Doxorubicin (DOX) serves as an anthracycline-based chemotherapy drug to treat multiple types of cancers and sarcomas, which include lung, breast, bladder, leukaemia, liver, head and neck cancers.

**Experimental approach:**

The electroanalysis of the trace level of DOX in this study was conducted using a Zn-Ni metal-organic framework nanosheets@graphene oxide nanocomposite (Zn-Ni MOF NSs@GO) after the modification of a screen-printed carbon electrode (Zn-Ni MOF NSs@GO/SPCE). Using chronoamperometry, cyclic voltammetry and differential pulse voltammetry, the electrochemical characteristics of the sensor as constructed for DOX measurement were examined.

**Key results:**

Consequently, the composite sensor demonstrated exceptional electrocatalytic performance in oxidizing DOX within a concentration range of 0.004 to 190.0 μM, with a detection limit of 0.001 μM. It was found that the Zn-Ni MOF NSs@GO/SPCE was effective at determining DOX in real samples, with good recovery values in the 97.1 to 102.0 % range.

**Conclusion:**

The designed sensor with cost-effective and good performance could be valuable for therapeutic drug monitoring and clinical diagnostics.

## Introduction

Cancer ranks as the most severe public health threat because it constitutes a permanent medical condition. Cancer has become the second most common cause of death, which follows cardiovascular disease as the leading cause. Chemotherapy represents a fundamental cancer treatment method in which doctors prescribe one or multiple medications to treat cancer through complete cancer elimination or cancer cell growth suppression [1,2]. The main factor determining drug quality control is testing various substances. Doxorubicin (DOX) serves as an anthracycline-based chemotherapy drug to treat multiple types of cancers and sarcomas, which include lung, breast, bladder, leukaemia, liver, head and neck cancers. The drug exerts its therapeutic action by binding to the DNA helix, thereby blocking transcription and replication in cancer cells via its anthracycline component [3-5]. The drug interacts with dissolved oxygen to generate reactive oxygen species that damage mitochondria. DOX produces several common chemotherapy side effects, which include nausea and vomiting, hair loss and weakened immunity, despite its high treatment effectiveness. The total cumulative dosage of DOX that a patient receives determines whether the drug will induce dangerous cardiotoxic side effects [6-8]. The procedure requires establishing specific medication dosages by monitoring DOX excretion and tracking drug concentrations in biological samples.

The detection of DOX has been achieved using various methods, including UV-Vis spectrophotometry [9], capillary electrophoresis [10], Raman spectroscopy [11] and high-performance liquid chromatography [12]. However, most of these techniques have numerous disadvantages, including high costs, large sample volumes, the need for highly qualified staff, time-consuming procedures, and complex sample pretreatment, which restricts their use to lab settings.

Because of their high sensitivity, low instrumentation costs, comparatively short analysis times, and the elimination of laborious extraction procedures, electrochemical techniques have become increasingly popular for pharmaceutical, environmental, and biological samples in recent years [13-17]. Screen-printed electrodes (SPE) and interdigital electrodes are two examples of planar substrates used to fabricate sensors. Among them, SPEs are more disposable than other conventional electrodes due to their tiny size, low cost, and light weight. As a result, SPEs are increasingly being used in the creation of electrochemical sensors [18-21].

In electrochemical methods, a problem arises with the working electrodes (*e.g.* low sensitivity or a short linear range) during sensor device fabrication. The reactivity and selectivity of electrochemical processes may be enhanced and exploited if the physicochemical structure of the interface (between the electrode surface and the solution) is regulated through thoughtful, sensible design. Investigating this area of study, which focuses on the investigation, description, and use of modified electrodes, more especially, the method by which altering an electrode's surface with species possessing particular characteristics can enhance the intended interface, was therefore deemed pertinent [22-26].

Nanomaterials have great promise for use in analytical chemistry as working-electrode modifiers because they exhibit distinctive mechanical, electrical, electronic, optical, magnetic, surface, and biological properties not found in traditional bulk materials [27-30].

Metal-organic frameworks (MOFs) and other porous crystalline structures made of both organic and inorganic components have become popular in recent years. Depending on the intended use, MOFs may be designed as a range of networks with varying chemical and physical properties due to the wide diversity of metal nodes and bridging ligands. MOFs can be used in a variety of applications, including drug delivery, energy storage, gas storage, colorimetric sensors, semiconductors, and more, due to their topological diversity, high specific surface areas, aesthetically pleasing structures, high porosity, and excellent chemical and thermal stability [31-34].

MOFs have emerged as novel electrode materials for electrochemical applications because their porous structure enables precise control of metal-ion electrochemical behaviour. The direct use of MOFs as electrode materials is limited because their mechanical stability is poor and their electrical conductivity is low [35]. The study results show that MOF-based composites exhibit greater stability and electrochemical performance than pure samples across multiple research fields [36]. Researchers have utilized graphene oxide (GO) and other readily available carbon substrates to enhance the conductive performance of MOFs by developing solutions that address existing challenges. The incorporation of MOFs into these materials addresses the widespread issue of graphene nanosheet agglomeration and significantly improves the electrical conductivity of the composite [37-40].

The research team developed a modified screen-printed carbon electrode (SPCE) that enables DOX detection using Zn-Ni MOF nanosheets@GO nanocomposites. The Zn-Ni MOF NSs@GO/SPCE sensor, therefore, showed increased electrocatalytic activity towards DOX. The voltammetric response of DOX showed a linear relationship with concentration over the range 0.004 to 190.0 μM, yielding a detection limit of 0.001 μM. The researchers assessed the practical use of the Zn-Ni MOF NSs@GO/SPCE sensor by testing DOX in real samples, achieving successful results.

## Experimental

### Chemicals and apparatus

All analytical-grade reagents were used exactly as provided, requiring no additional purification. Orthophosphoric acid was used to prepare phosphate buffer solutions (PBS), which served as the electrolyte supporting the electrochemical measurement of DOX over the pH range 2.0 to 9.0. The researchers used a NaOH solution to adjust the pH.

The electrochemical tests were conducted on an Autolab potentiostat/galvanostat system (PGSTAT-302 N, Netherlands). The GPES software functioned as the control system for all experimental parameters. The screen-printed three-electrode configuration (DropSens, DRP-110, Spain) consisted of a Zn-Ni MOF NSs@GO/SPCE working electrode, a carbon auxiliary electrode and a silver pseudo-reference electrode. The pH was determined using a Metrohm 710 pH meter. The Zn-Ni MOF NSs@GO nanocomposites were synthesized based on their previous work [41].

### Zn-Ni MOF NSs@GO modified-screen-printed carbon electrode

The Zn-Ni MOF NSs@GO/SPCE was initially prepared by dispersing 1.0 mg of the Zn-Ni MOF NSs@GO nanocomposite in 1.0 mL of deionized water using ultrasonic agitation to create a homogeneous suspension. After casting 2.0 μL of the homogeneous suspension onto the SPCE surface, the solvent was allowed to evaporate at room temperature. The Zn-Ni MOF NSs@GO/SPCE was used as the working electrode. The electrochemical surface areas of Zn-Ni MOF NSs@GO/SPCE and bare SPCE was calculated to be 0.060 and 0.0134 cm^2^, respectively.

### Preparation of injection sample of doxorubicin

For investigating the ability of the *Zn-Ni MOF NSs@GO/SPCE* sensor in the analysis of pharmaceutical formulations, 1.0 ml of DOX HCl injection solution (vial 25 ml contains 50 mg DOX·HCl) was diluted 10 times using PBS without any other pretreatment. Then, a certain volume of the diluted sample was transferred to a 25 ml volumetric flask, which was filled to the mark with PBS. This sample was analysed using differential pulse voltammetry (DPV) to determine the DOX concentration in the diluted sample. Subsequently, the diluted samples were separately spiked with various concentrations of DOX solution. The DPVs of these samples were then recorded.

## Results and discussion

### Electrochemical behaviour of doxorubicin at the Zn-Ni MOF NSs@GO/SPCE

The oxidation of DOX in PBS solution depends on pH. CV measurements were conducted to investigate how different pH levels in 0.1 M PBS, ranging from 2.0 to 9.0, affected DOX oxidation at the Zn-Ni MOF NSs@GO/SPCE. The CV data show that DOX exhibits its highest anodic peak current at pH 7.0, which decreases as pH increases. Therefore, PBS buffer at pH 7.0 was identified as the optimal pH for DOX detection using the Zn-Ni MOF NSs@GO/SPCE system.

**Figure 1.**
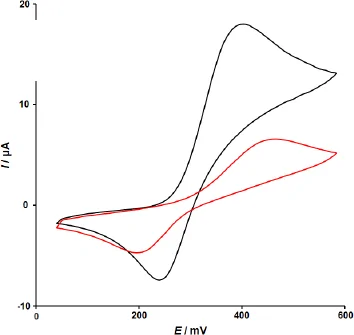
CV curves of 100.0 μM DOX in 0.1 M PBS (pH 7.0) by bare SPCE (red curve) and Zn-Ni MOF NSs@GO /SPCE (black curve) at scan rates of 50 mV s^-1^

The Zn-Ni MOF NSs@GO/SPCE showed better sensing performance for DOX 100.0 μM when tested against the bare SPCE electrode (Figure 1). The Zn-Ni MOF NSs@GO/SPCE exhibits separate oxidation and reduction peaks at 400 and 240 mV, while the SPCE bare displays a broad oxidation peak that peaks at 470 mV with a reduction peak at 200 mV. The Zn-Ni MOF NSs@GO nanocomposite shows catalytic activity, evidenced by increased DOX oxidation peak current and a shift of the oxidation peak potential towards more negative values.

### Influence of scan rate

**Figure 2.**
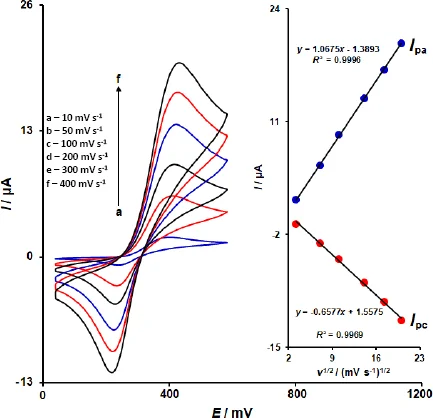
Zn-Ni MOF NSs@GO/SPCE CV curves of 30.0 μM DOX in 0.1 M PBS (pH 7.0) at different scan rates from 10 to 400 mV s^-1^. Inset: The linear relationship between redox currents (*I*_pa_ and *I*_pc_) and the *v*^1/2^

In CV, the relationship between peak current and scan rate (*v*) typically provides useful information about the electrochemical process. Consequently, CV was used to examine how scan rates affected the DOX peak currents at the Zn-Ni MOF NSs@GO/SPCE in 0.1 M PBS (pH 7.0) (Figure 2).

Consequently, Figure 2 also displays the electrochemical behaviour of 30.0 μM DOX at various scan rates ranging from 10 to 400 mV s^-1^. The scan rates and the peak current heights (*I*_pa_ and *I*_pc_) have a strong linear relationship. Additionally, there is a minor variation in the peak potentials (both *E*_pa_ and *E*_pc_) with potential scan rates. Figure 2 (Inset) shows a linear relationship between the square root of the scan rate (*v*^1/2^) and peak current heights (*I*_pa_ and *I*_pc_). This suggests that a diffusion mechanism governs DOX redox process at the Zn-Ni MOF NSs@GO/SPCE.

### Chronoamperometric studies

**Figure 3.**
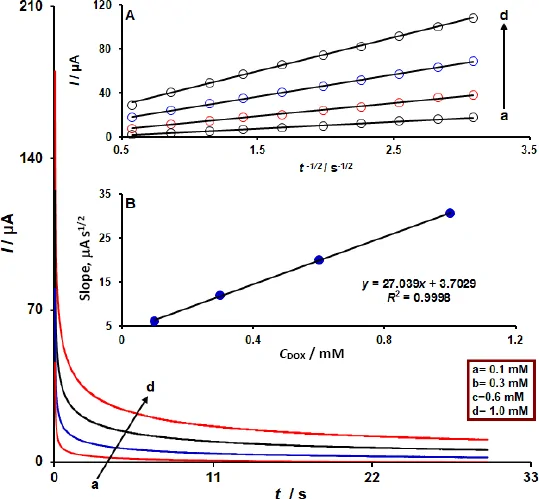
Chronoamperograms for various DOX concentrations ranging from 0.1 to 1.0 mM acquired at Zn-Ni MOF NSs@GO/SPCE in 0.1 M PBS (pH 7.0). (A) Plots of *I* versus *t*^-1/2^ is shown as an inset. (B) A plot of the straight-line slopes against the concentration of DOX

By setting the working electrode potential at 440 mV for the different concentrations of DOX (0.1 to 1.0 mM) in 0.1 M PBS (pH 7.0), chronoamperometric analyses of DOX at Zn-Ni MOF NSs@GO/SPCE were carried out (Figure 3). The Cottrell equation (*I* = *nFAD*^1/2^*C*_b_*π*^-1/2^*t*^-1/2^) describes the current measured for the electrochemical reaction under the mass transport constrained condition for an electroactive substance (in this example, DOX) with a diffusion coefficient of *D*. The best fits for various DOX doses were found using experimental plots of *I*_p_ *vs. t*^-1/2^ (Figure 3A). Plotting the slopes of the obtained straight lines against DOX concentration was the next step (Figure 3B). From the resulting slope and Cottrell equation, the mean value of the *D* was found to be 6.24×10^-5^ cm^2^ s^-1^.

### Quantitative measurements of doxorubicin at Zn-Ni MOF NSs@GO /SPCE sensor using differential pulse voltammetry

**Figure 4.**
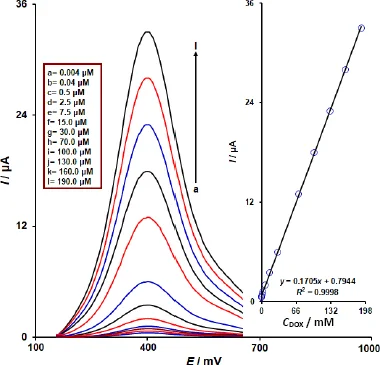
Zn-Ni MOF NSs@GO/SPCE in PBS (0.1 M; pH 7.0) with varying DOX concentrations (0.004 to 190.0 μM by DPVs. Plot of the *I*_pa_ *vs.* DOX concentrations is shown in the inset

The DPV response of the Zn-Ni MOF NSs@GO/SPCE sensor to DOX was examined by varying the DOX concentration from 0.004 to 190.0 μM. The calibration plot shows a linear segment from 0.004 to 190.0 μM, and Figure 4 shows that the peak current increases with DOX concentration. The related linear regression equation is *I*_pa_ = 0.1705 *C*_DOX_ + 0.7944 (*R*^2^ = 0.9998), with a sensitivity of 0.1705 μA μM^-1^. The limit of detection (LOD) was determined to be 0.001 μM using the formula LOD = 3*S*_b_/*m*, where *S*_b_ is the blank standard deviation and *m* is the calibration curve's slope.

### Application of the Zn-Ni MOF NSs@GO /SPCE platform for doxorubicin analysis in real sample

To verify the use of Zn-Ni MOF NSs@GO/SPCE, the suggested sensor's applicability and reliability in real samples were examined. The DPV technique was used to determine DOX in DOX injection samples after sample preparation and appropriate dilution. Table 1 provides a summary of the analysis's findings. The test recoveries fell between 97.1 and 102.0 % categories. Zn-Ni MOF NSs@GO/SPCE may be effectively employed to determine DOX in doxorubicin injection samples, according to the recovery findings shown in Table 1.

**Table 1.** Application of the proposed method to the determination of DOX in DOX injection at the Zn-Ni MOF NSs@GO/SPCE (*n* = 5)

DOX concentration, μM		Recovery, %	RSD, %
Added	Found		
0	2.9	-	3.3
2.0	5.0	102.0	1.9
4.0	6.7	97.1	2.2
6.0	9.0	101.1	2.7
8.0	10.8	99.1	2.3

## Conclusion

In conclusion, the sensing platform for detecting the anti-cancer medication DOX was successfully designed using Zn-Ni MOF NSs@GO/SPCE. The Zn-Ni MOF NSs@GO/SPCE demonstrated a greater electrochemical response and electro-catalytic activities toward the determination of DOX in comparison to the bare SPCE because of the porous structure and large surface area of Zn-Ni MOF, the excellent conductivity of GO, and the synergistic interaction of those two materials. With a low LOD of 0.001 μM, the Zn-Ni MOF NSs@GO/SPCE sensor demonstrated exceptional sensitivity for DOX in the 0.004 to 190.0 μM linear concentration range. Ultimately, good recovery data were collected and the suggested approach showed promise in determining DOX in actual samples.
